# Resting-State Temporal Synchronization Networks Emerge from Connectivity Topology and Heterogeneity

**DOI:** 10.1371/journal.pcbi.1004100

**Published:** 2015-02-18

**Authors:** Adrián Ponce-Alvarez, Gustavo Deco, Patric Hagmann, Gian Luca Romani, Dante Mantini, Maurizio Corbetta

**Affiliations:** 1 Center for Brain and Cognition, Computational Neuroscience Group, Department of Information and Communication Technologies, Universitat Pompeu Fabra, Barcelona, Spain; 2 Institució Catalana de la Recerca i Estudis Avançats (ICREA), Universitat Pompeu Fabra, Barcelona, Spain; 3 Department of Radiology, Lausanne University Hospital and University of Lausanne (CHUV-UNIL), Lausanne, Switzerland; 4 Signal Processing Lab 5, Ecole Polytechnique Fédérale de Lausanne (EPFL), Lausanne, Switzerland; 5 Institute of Advanced Biomedical Technologies—G. d’Annunzio University Foundation, Department of Neuroscience and Imaging, G. d’Annunzio University, Chieti, Italy; 6 Department of Health Sciences and Technology, ETH Zurich, Zurich, Switzerland; 7 Department of Experimental Psychology, University of Oxford, Oxford, United Kingdom; 8 Department of Neurology, Radiology, Anatomy of Neurobiology, School of Medicine, Washington University in St. Louis, St. Louis, Missouri, United States of America; Hamburg University, GERMANY

## Abstract

Spatial patterns of coherent activity across different brain areas have been identified during the resting-state fluctuations of the brain. However, recent studies indicate that resting-state activity is not stationary, but shows complex temporal dynamics. We were interested in the spatiotemporal dynamics of the phase interactions among resting-state fMRI BOLD signals from human subjects. We found that the global phase synchrony of the BOLD signals evolves on a characteristic ultra-slow (<0.01Hz) time scale, and that its temporal variations reflect the transient formation and dissolution of multiple communities of synchronized brain regions. Synchronized communities reoccurred intermittently in time and across scanning sessions. We found that the synchronization communities relate to previously defined functional networks known to be engaged in sensory-motor or cognitive function, called resting-state networks (RSNs), including the default mode network, the somato-motor network, the visual network, the auditory network, the cognitive control networks, the self-referential network, and combinations of these and other RSNs. We studied the mechanism originating the observed spatiotemporal synchronization dynamics by using a network model of phase oscillators connected through the brain’s anatomical connectivity estimated using diffusion imaging human data. The model consistently approximates the temporal and spatial synchronization patterns of the empirical data, and reveals that multiple clusters that transiently synchronize and desynchronize emerge from the complex topology of anatomical connections, provided that oscillators are heterogeneous.

## Author Summary

The spontaneous or resting-state activity of the brain is organized into multiple spatial patterns of correlated activity. These patterns have been associated with functional interacting brain networks. Recent studies show that the correlations among brain regions are not stationary, but evolve over time, and have refocused the study of spontaneous brain activity on characterizing these time-varying functional interactions. In this article, we show that the synchrony between the BOLD activities of different brain regions displays global slow fluctuations that reflect the dynamical association and dissociation of functional synchronized clusters. Using a network of anatomically connected phase oscillators, we show that transiently synchronized patterns emerge from the interplay between nonlinear dynamics and the complex, but static, network topology. Our results suggest that the brain constantly explores its dynamical repertoire during rest, which allows for an all-around visitation of functional states.

## Introduction

The spontaneous activity of the brain is organized into multiple spatial patterns of correlated activity across different brain regions, known as ‘resting-state networks’ (RSNs) [1–12]. The topography of the resting-state networks overlaps with functional networks observed during cognitive load, including the default mode network, the fronto-parietal network, and other attention, visual, auditory, and sensorimotor networks [13–16]. The temporal evolution of resting-state activity has only recently been subject to investigation. Recent studies have demonstrated that the correlations among brain regions, both within and between networks, evolve over time [11, 17–20]. These results suggest that spatial patterns are formed, dissolved, and reformed over time, so that resting activity can be divided into subsets or “communities” of brain regions that strongly interact for a period of time. Importantly, time-varying functional connectivity has been reported both in awake humans and anesthetized macaques [20], thus the temporal variability of functional interactions is not likely to be produced by transitions between different mental states, but it may be an emergent property of the complex brain network.

These results suggest that the interplay between space and time dimension is crucial to unravel the mechanistic origins of spontaneous activity. How non-stationary functional connectivity emerges in the brain network remains an open question though. Current large-scale models of spontaneous activity have been built to approximate the averaged functional connectivity and assume that the stationary long-range functional correlations rise from the interplay between the underlying anatomical connectivity structure and the neural dynamics [21–24]. It is unclear what mechanisms generate the temporal fluctuations in functional connectivity. Are the time-varying interactions governed by the topology of the brain anatomical connectivity? Or do they reflect the variation of the structural connections due to interplay between the dynamical states and the network couplings, such as, for example, in the case of short term synaptic plasticity?

In the present study we used phase synchronization to measure, with enhanced temporal resolution, the time-varying functional interactions of the resting-state fMRI BOLD signals of human subjects. This approach has been successfully applied to the study of fMRI data during rest and during viewing of natural scenes [25–27]. We found that global oscillations and multiple transient synchronized clusters (i.e. communities) are present in the data. By studying a network of phase oscillators in which connections are given by the brain’s anatomical connectivity, we showed that transiently synchronized networks emerge from the topology of anatomical connections and the heterogeneity among oscillators.

## Results

### Dynamics of phase synchronization of resting-state BOLD signals

We analyzed the BOLD activity from a total of 48 scanning sessions, from 24 healthy human subjects, of 600s (sampled in *T* = 300 frames) during resting-state condition, and acquired using standard fMRI techniques (see Methods). The dataset consists of whole-brain BOLD signals, averaged over *n* = 66 cortical brain regions (see S1 Table).

**Fig 1:**
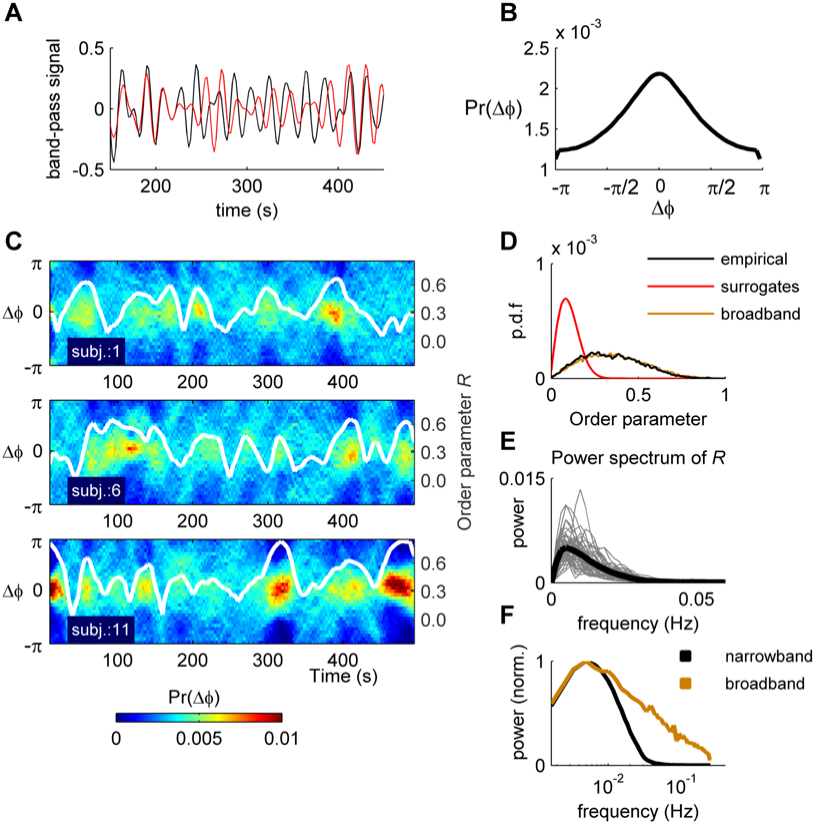
Temporal dynamics of phase interactions. **a)** Example narrowband signals from two different brain regions (rPARH and rFUS). **b)** Probability density function of the phase differences, Pr(Δφ), across all pairs of brain regions and all time steps. **c)** Temporal evolution of Pr(Δφ), for 3 example scanning sessions from different subjects. The color code indicates the values of Pr(Δφ) at each time step (see the color bar at the bottom).White trace: Time course of the order parameter *R*(*t*) (right y-axis). **d)** Probability density function of the order parameter values for all sessions (black) compared to phase-randomized surrogates (red) and broadband signals (brown). **e)** Power spectrum of the order parameter. Gray traces: single scanning sessions; black trace: mean across all sessions. **f)** Comparison of the averaged power spectrum of the order parameter for narrowband (black) and broadband signals (brown). Power spectra were normalized by the corresponding maximum value.

The interaction between BOLD signals of different brain regions was measured using the instantaneous phase synchronization. For obtaining the phase of each brain region, the signals were first band-pass filtered within the narrowband 0.04–0.07Hz. Previous work has shown that this frequency band contains more robust and functionally relevant signals than the other bands [26]. Moreover, narrowband filtering is a methodological requirement for obtaining meaningful signal phases [26]. As shown in the Methods section, phase interactions fairly describe the interactions among the narrowband signals and, as shown in the following, they allow for a time-resolved analysis of interactions (see also S1 Fig.). As shown in Fig. 1a, the phase relation between two given BOLD narrowband signals changes over time, alternating periods during which the oscillations are in-phase and periods during which the oscillations are out-of-phase. To quantify these phase relations we computed the instantaneous phase φ_*k*_(*t*) of each narrowband signal *k* using the Hilbert transform (HT) (see Methods). After this, the first and last 10 time steps were discarded to avoid border effects inherent to the HT, so that in the following *T* = 280. We next calculated the phase difference, Δφ_*kl*_(*t*) = φ_*k*_(*t*)–φ_*l*_(*t*), for each pair of brain regions *k* and *l*, at each time step *t*. Across all pairs of signals and time steps, the probability density function (p.d.f.) of the pairwise phase differences, noted Pr(Δφ), is centred on zero (Fig. 1b). However, Pr(Δφ) is not constant, but evolves over time, going from a uniform distribution (i.e., the phases are independent) to a distribution that is densely concentrated around zero (i.e., high phase synchrony) (Fig. 1c, color plots).

To describe the temporal evolution of Pr(Δφ) we computed the order parameter, $R(t)=\left|\sum_{k=1}^{n}e^{j\varphi_{k}(t)}\right|/n$, that measures the global level of synchronization of the *n* oscillating signals. Under complete independence, the *n* phases are uniformly distributed and thus *R* is nearly zero, whereas *R* = 1 if all phases are equal. The temporal evolution of *R*(*t*) effectively tracts the evolution of Pr(Δφ) (Fig. 1c, white traces). Phase synchronizations were significantly higher (p<10^–10^, t-test) than the expected accidental synchronizations of phase shuffled surrogates, designed to decorrelate the phases while preserving the power spectrum of the original signals (Fig. 1d) (see Methods). Notably, the power spectrum of *R* shows that the global level of synchronization evolves on an ultra-slow (<0.01 Hz) time-scale (Fig. 1e). The peak frequency averaged across all scanning sessions is equal to 0.006±3.10^–4^ Hz, i.e., one order of magnitude slower than the frequency of the narrowband signals (comprised between 0.04–0.07 Hz). This also robustly observed in individual scanning sessions for which the peak frequency of *R* ranges between 0.003–0.010 Hz. Finally, we evaluated the statistics of the order parameter *R* obtained using the broadband signals. We found that both the distribution of *R* and the peak frequency of its time evolution was preserved using the broadband signals (Fig. 1d, f), indicating that the observed slow fluctuations of the global synchronization are not a product of the narrowband filtering.

### Transient activation of functional synchronization networks

In the following, we show that the spatiotemporal patterns of synchronization reflect the formation and break up of various different clusters of synchronized brain regions. To show this, we used a decomposition technique, recently proposed for the detection of communities in time-varying networks [28], to represent both the topology of synchronized clusters and their activation over time.

**Fig 2:**
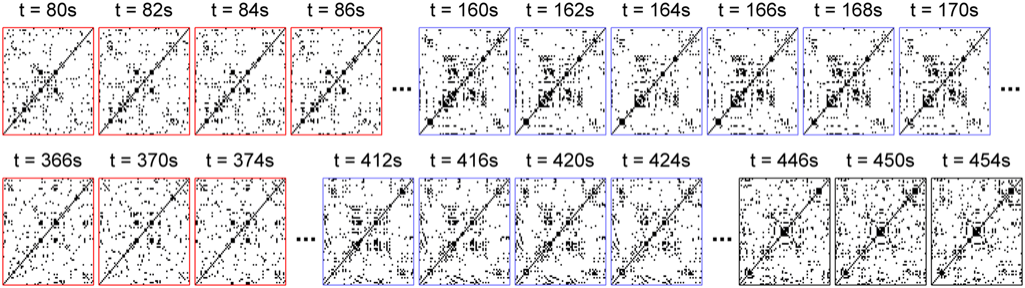
Spatiotemporal synchronization patterns. A synchronization matrix **Q** was built at each time step *t* (*t* = 1, .., *T*) by calculating the phase difference of each pair of empirical analytic signals and imposing a synchronization threshold: Q_*ij*_(*t*) = 1 if |*φ*_*j*_(*t*)-*φ*_*i*_(*t*)|<π/6 and Q_*ij*_(*t*) = 0 otherwise (1 *≤ i*, *j ≤ n*). The temporal evolution of the synchronization matrix **Q** is shown for an example scanning session (session #3) and for selected time periods; entries equal to one are represented in black, null entries are represented in white. The synchronization patterns framed in *blue* and *red* last several seconds and reoccur during disjointed time periods.

**Fig 3:**
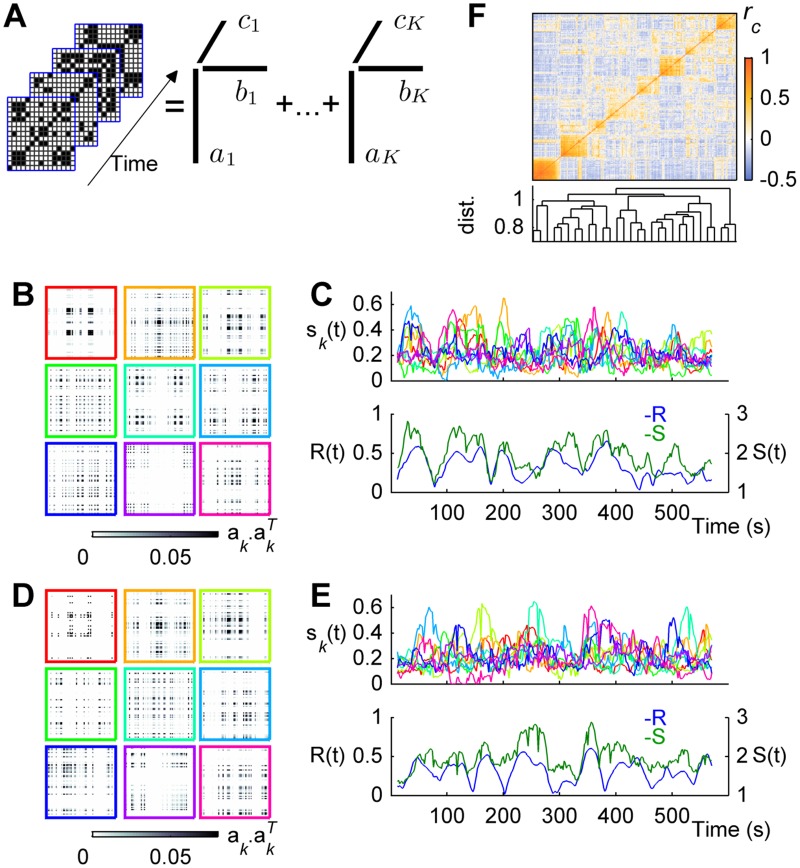
Community structure of spatiotemporal synchronization patterns. **a)** The temporal evolution of the synchronization matrix is represented in a *n×n×T* tensor **T**. The tensor can be factorized as a sum of *K* rank-one tensors, each one being an outer product of three vectors, ***a***_***k***_, ***b***_***k***_, ***c***_***k***_, of dimension equal to *n*, *n*, and *T*, respectively (Equation 8). The network communities are contained in the vectors ***a***_***k***_, the elements of which give the participation weight of each node (i.e., brain region) in the community *k*. The temporal activation *s*_*k*_(*t*) of each community *k* is related to ***c***_***k***_ and to the participation weights as: *s*_*k*_(*t*) = *c*_*k*_(*t*)Σ_*j*_ *a*_*k*_(*j*). **b)** Detected community patterns (***a***_***k***_. ***a***_***k***_^***T***^) for the example session #1. **c)** *Top*: temporal activation strength of each community, for the scanning session #1 (same colors as in (b)). *Bottom*: temporal evolution of both the order parameter *R*(*t*) (left y-axis) and the total activation strength [*S*(*t*) = Σ_*k*_ *s*_*k*_(*t*)] (right y-axis), for session #1. **d-e)** same as (b-c) but for session #7. **f)** Correlation matrix between all detected communities from all scanning sessions (*top*), re-arranged according to cluster membership. *Bottom*: corresponding dendrogram based on correlation coefficients.

Specifically, we reduced the spatiotemporal distribution of the *n* phases into a binary three-dimensional matrix, or tensor, of size *n*×*n*×*T*, for each scanning session. At each time step *t*, a symmetric *n×n* synchronization matrix was constructed, with (*i*, *j*) elements equal to 1 if |*φ*_*j*_(*t*)*-φ*_*i*_(*t*)|<π/6 and 0 otherwise (1 *≤ i*, *j ≤ n*). The synchronization matrix evolved in time and displayed different topological patterns that last over several seconds and reoccur over time (Fig. 2). To detected these patterns, each constructed tensor was decomposed into a sum of *K* rank-one tensors, or “components”, using a non-negative tensor factorization (NNTF) technique, a higher-order analogue of Principal Component Analysis (see Methods) (Fig. 3a). Each of the *K* components is a rank-one tensor, namely an outer product of 3 vectors, ***a***_***k***_, ***b***_***k***_, and ***c***_***k***_—in our case, due to symmetry we have: ***a***_***k***_ = ***b***_***k***_. This decomposition separates space and time: the *n* elements of ***a***_***k***_ give the participation weight of each node in the component *k*, i.e. the community structure of component *k*, while the *T* elements of ***c***_***k***_ relate to the activation level of component *k* at time step *t*, i.e. the activation of community *k* over time.

For each scanning session we detected the communities and associated activations (Fig. 3b-e). The estimated number of components range between 6–13 for the individual scanning fMRI sessions and in the following, for simplicity, we chose the median value (*K* = 9) for all scanning sessions (see Methods). We found that similar community patterns were present in different scanning fMRI sessions (Fig. 3b, d). This is clearly shown by the modular structure of the correlation matrix between all pairs of components of all sessions (Fig. 3f), rearranged according to cluster membership (cophenetic correlation coefficient: 0.69). Communities are transiently activated over time (Fig. 3c, e, *top*), such that, most of the time, the synchronization matrix is closer to one community or a superposition of few communities. The averaged duration of activation (i.e., *s*_*k*_>0.1, see Methods) across all communities is 11.80±0.20s. Moreover, we found that the sum of activations of all communities, noted *S*(*t*), consistently mirrors the temporal evolution of the order parameter (Fig. 3c, e, *bottom*), the correlation coefficient between *S*(*t*) and *R*(*t*) averaged over all sessions being 0.76 ± 0.03. These results suggest that switching among multiple synchronized clusters that last ~10 seconds underlie the temporal evolution of phase synchronization.

**Fig 4:**
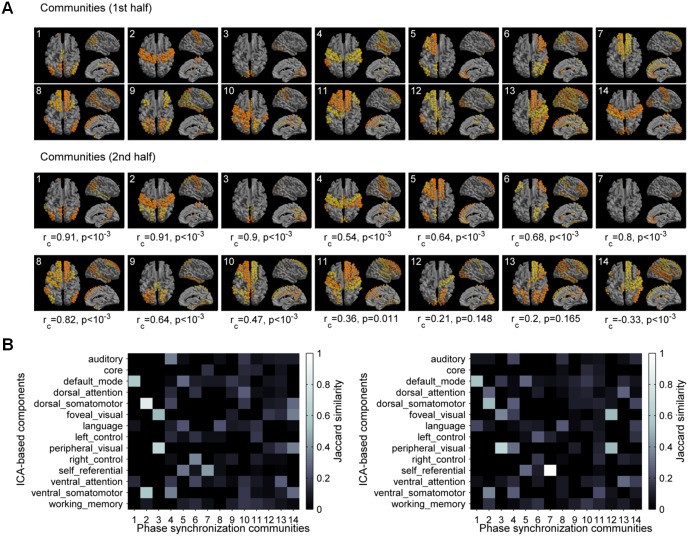
Topology of the synchronization communities. **a)** Spatial organization of the communities obtained with the first half of the sessions (*top*) and the second half of the sessions (*bottom*). For each community, the brain regions with the highest participation weights are presented (yellow: 0.1<*a*_*k*_(*i*)<0.2; orange: *a*_*k*_(*i*)>0.2). The community patterns of the second half of the sessions were matched to the ones of the first half of the sessions (below each panel the correlation between the community *i* from the first half-dataset and the community *i* from the second half-dataset is presented; *r*_*c*_: correlation coefficient; *p*: p-value). **b)** The synchronization communities were compared to the resting-state networks obtained using ICA. As a measure of similarity we used the Jaccard index. The Jaccard similarity matrix between ICA-based components and synchronization communities is shown for the first half of the data (*left*) and the second half of the data (*right*). The spatial patterns given by the synchronization communities include: the default mode network (DMN, community 1), the somato-motor network (2), the visual network (3), the auditory/somato-motor network (4), the self-referencial/DMN network (5), the right cognitive control network (6), and other networks (7–14) that are overlaps of the previous ones and other functional networks detected using ICA.

We further examined the topological organization of the communities across all scanning sessions. For this population analysis, we concatenated in time the tensors of the scanning sessions. To test robustness of the detected communities we divided the dataset in two halves. The selected number of components was equal to 14 for the two tensors constructed by concatenating each half of all scanning sessions, respectively (see Methods). We found that most of the communities are consistently present in both half-datasets (Fig. 4a). Furthermore, we found that the communities represent functional networks, including the default mode network, the somato-motor network, the visual network, the auditory network, the cognitive control networks, the self-referential network, and combination of these and others RSNs. This can be better appreciated by comparing the synchronization communities with the spatial components obtained using spatial Independent Component Analysis (ICA) on the same data (see Methods). We found that the synchronization communities project to specific ICA RSNs or combinations of RSNs (Fig. 4b), i.e., some communities project to one ICA RSNs, in particular the default-mode network, while others clearly show combination of different ICA RSNs. Such combinations suggest that activations of synchronization communities may be seen as concomitant activations of multiple RSNs. The results were consistent using the communities extracted from the two half-datasets.

To test the effect of using a different synchronization threshold for constructing the binary synchronization tensors, we compared the obtained communities (i.e., the vectors *a*_*k*_) with the threshold equal to π/4 to the ones obtained previously with the threshold equal to π/6 (S2 Fig.). We found that similar community patterns are consistently found for different synchronization thresholds.

### Emergence of transient synchronized clusters in an anatomically-constrained network of oscillators

To get further theoretical insights on the emergence of transiently synchronized networks among segregated brain regions, we examined a model of oscillators interconnected through the brain anatomical connections estimated using diffusion imaging on human subjects (see Methods). Here, we used a version of the Kuramoto model, which is the canonical model for studying synchronization phenomena [29]. Within this model, each node of the network is model by a phase oscillator, with an intrinsic frequency *ω*_*i*_ in the 0.04–0.07Hz band (*i* = 1, …, *n*). The intrinsic frequencies were estimated from the data, as given by the averaged peak frequency of the narrowband BOLD signals of each brain region. The state of each oscillating node *i* is determined by its phase, *φ*_*i*_(*t*), and the interaction between nodes depends both on the structural couplings and the phase difference between the nodes. The model has one single parameter, *G*, that represents the global scaling of the anatomical connectivity matrix.

**Fig 5:**
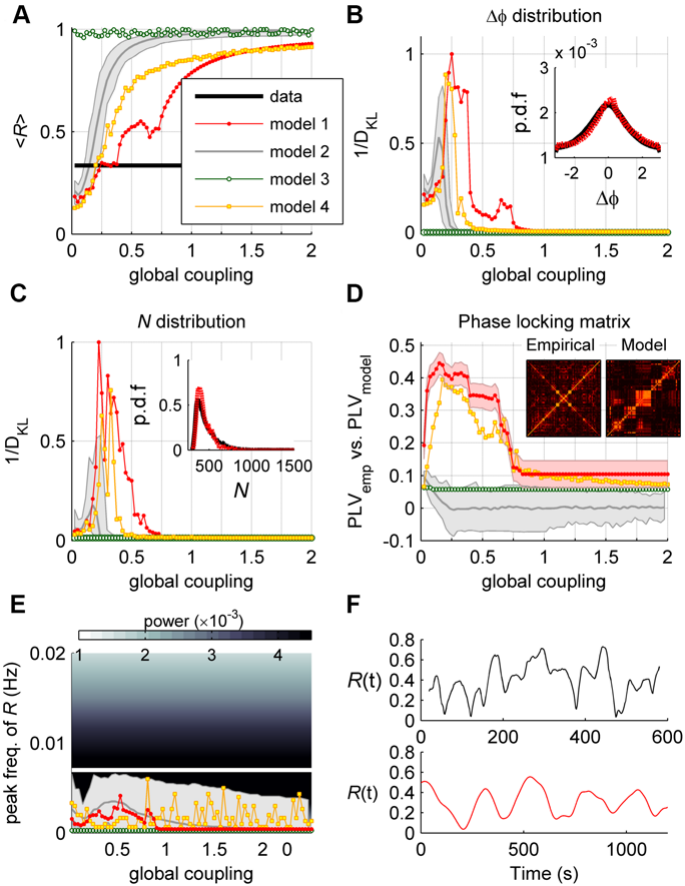
An anatomically-constrained network of phase oscillators approximates the empirically observed phase statistics. The empirical statistics (black) were compared to the statistics generated by the anatomically connected heterogeneous model (model 1, *red*) and three control models: 100 realizations of the heterogeneous model with shuffled connectivity (model 2, *gray*, mean ± 95% confidence interval across realizations (gray area)), the anatomically connected homogeneous model (model 3, *green*), and the anatomically connected homogeneous stochastic model (model 4, *yellow*), with noise amplitude σ = 0.2. **a)** Averaged value of the order parameter, <*R*>. **b)** Similarity (1/*D*_*KL*_) between the phase differences distribution, Pr(Δφ), of the empirical data and of each model. All similarity values were normalized to the maximum similarity for model 1. *Inset*: Pr(Δφ) from the empirical data (*black*) and from model 1 (*red*) generated with the best-fit parameter. **c)** Similarity (1/*D*_*KL*_) between the distribution of the number *N* of synchronized pairs, Pr(*N*), of the empirical data and each model (normalized by the maximum similarity for model 1). *Inset*: Pr(*N*) distribution from the empirical data (*black*) and from model 1 (*red*) generated with the best-fit parameter. **d)** Agreement (correlation) between the empirical PLV matrix and the models’ PLV matrices. Red area: 95% confidence interval of the sample Pearson correlation coefficient. *Insets*: PLV matrix from the empirical data and from model 1 generated with the best-fit parameter. **e)** Peak of the power spectrum of *R*. The color code represents the empirical frequency power of *R* and the white line represents the empirical peak frequency. **f)** *Top*: time evolution of *R* for one single fMRI session. *Bottom*: time evolution of the *R* of model 1 for *G* = 0.2. Note different time-scales (x-axis).

The model dynamics were compared to the empirical data, for varying values of *G*. As *G* increases the mean order parameter <*R*> goes from the independent scenario (<*R*>*≈n* ^-1/2^) to a scenario in which all oscillators are synchronized, and for *G* ranging from 0.2 to 0.3 the model reproduces the value observed in the empirical data, equal to <*R*> = 0.335 ± 0.016 (Fig. 5a). Within this parameter range, the model maximally approximates the distribution of phase differences Pr(Δφ) (Fig. 5b)—the fitting of the distribution was evaluated using the inverse of the Kullback-Leibler divergence (1/*D*_*KL*_) between distributions (see Methods). Moreover, also within this parameter range, the model maximally approximates the distribution of synchronized pairs of nodes, i.e., the distribution of the number *N*(*t*) of (*i*, *j*) pairs such that |*φ*_*j*_(*t*)-*φ*_*i*_(*t*)|<π/6, noted Pr(*N*) (Fig. 5c). In addition to the previous global phase statistics of the system of oscillators, we also test whether the model replicates the detail phase relations between oscillators, as given by the matrix of phase locking values (PLV, see Methods). We compared the PLV matrices of the model and the empirical data by calculating the Pearson correlation coefficient between corresponding elements of the upper triangular part of the two matrices. We found that the maximal agreement between the model and the data is achieved for the parameter range *G* = 0.1–0.3 (Fig. 5d). Finally, we examined the temporal evolution of phase synchronization by computing the peak frequency of the order parameter. We found that, for all values of *G*, the order parameter fluctuates slowly over time, with a frequency comprised between 43.10^-5^–47.10^-5^ Hz (Fig. 5e-f). Although this frequency range is lower than the one empirically observed, the model replicates an important feature of the data, namely that fluctuations of the order parameter are one order of magnitude slower than the frequencies of the oscillators. Hence, there exists a range of the parameter *G* for which the model consistently approximates several global and detailed spatiotemporal phase statistics of the empirical data.

We next examined which features of the above model (model 1) contribute to the generation of the different statistics. We first perturbed the topology of the anatomical connectivity matrix by generating surrogate connectivity matrices, the elements of which were taken randomly from the original distribution of connection weights, while keeping the distribution of *ω* as in model 1. This model (model 2) fairly approximates both <*R*> and Pr(Δφ), but it leads to significantly lower prediction of Pr(*N*) compared to model 1 and cannot fit the PLV matrix (Fig. 5a-e, gray trace). This indicates that the topology of the connectivity is an important feature of the model to approximate the observed phase statistics.

Second, we keep the connectivity as in model 1 but imposed homogeneity in the intrinsic frequencies *ω* by setting its value to 0.05 Hz for all nodes (model 3). Homogeneity leads to the trivial state of complete synchronization of all oscillators, thus destroying the rich dynamics observed in the original model (Fig. 5a-e, green trace). Thus, heterogeneity is also a key ingredient of the model for predicting the observed phase statistics.

Finally, we tested the alternative scenario in which heterogeneity is produced by stochastic forces. Noise is ubiquitous in neural systems and plays an important role in neural dynamics [30], it is thus important to examine whether the observed phase statistics can be modeled by noise-driven dynamics. For this, we imposed homogeneity in the intrinsic frequencies as above and we added uncorrelated white noise to the model (model 4). For sufficiently small global couplings, the noise prevents the network from reaching full synchrony. This control model consistently predicts <*R*>, Pr(Δφ), and the PLV matrix, but it leads to a lower prediction of Pr(*N*) and to slower variations (<2.10^–3^ Hz) of the order parameter than in model 1 (Fig. 5a-e, yellow trace). The same results were obtained in an exhaustive analysis in the {*G-σ*} plane, where *σ* is the noise intensity (see S3 Fig.). Thus, the deterministic model, for which transient synchronization arises as a consequence of heterogeneity in natural frequencies, explains the empirical observations better than the model with noise. This indicates that the dynamics are less likely to be noise-driven than to be produced by intrinsic heterogeneities in the nonlinear deterministic system. In conclusion, both topology and heterogeneity in the model are essential to approximate the statistics observed empirically.

We next tested whether the previous results are also found in frequency bands other than 0.04–0.07 Hz. For this, we band-pass filtered the fMRI data in different narrow frequency bands within 0.01 and 0.13 Hz, we calculated the phase statistics for each frequency band, and we tested the predictive power of the Kuramoto model using the corresponding distribution of intrinsic frequencies and the anatomical connectivity. We found that the model consistently approximates the empirical phase statistics within the same parameter range (*G* = 0.1–0.3) (S4 Fig.).

**Fig 6:**
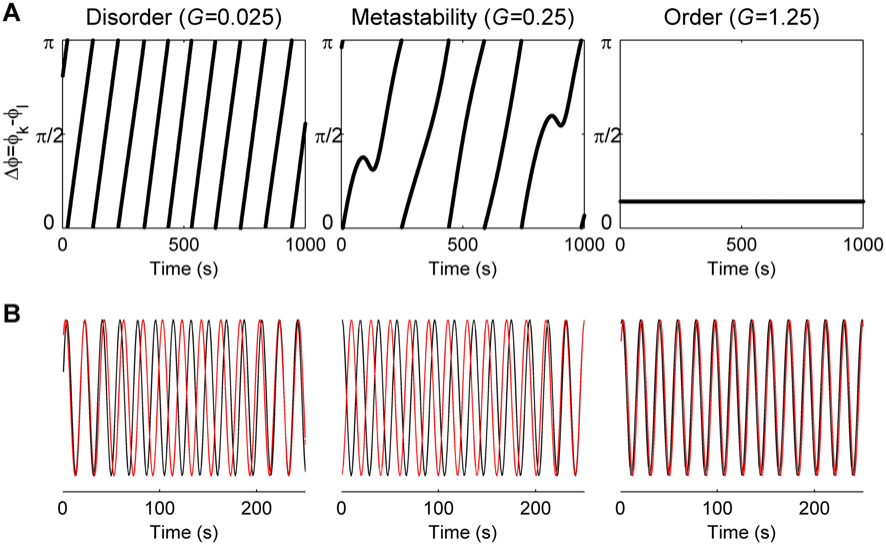
Dynamical range of the anatomically-constrained phase oscillators’ network. **a)** Time evolution of the phase difference between two nodes of the anatomically-connected heterogeneous Kuramoto model, for three values of the global coupling *G*. *Left*: in the weakly connected case (*G* = 0.025) the phases run almost independently; *middle*: with moderate coupling (*G* = 0.25) the phases tend to lock for short periods of time, as revealed by the deflections in the trajectory of the relative phase, indicating the presence of metastability; *right*: with strong coupling (*G* = 1.25) the phases are locked. **b)** Corresponding oscillations of the two nodes, for the three dynamical regimes.

Altogether, the above results show that the empirical statistics are well described in the region of partial synchronization, the parameter range between disorder (asynchrony) and complete order (full synchrony). This parameter region is characterized by metastability (Fig. 6). Indeed, it has been argued that step-like increases of the order parameter as a function of the global coupling is an indication of metastability [31]. This is clearly shown by the behavior of the relative phase between two given nodes of the network model (Fig. 6a). For small values of the global coupling (*G*), the phases run practically independently. For intermediate values of *G*, quasi-phase-locking events are indicated by the deflections of the relative phase’s trajectory. Such events appear transiently and are separated by periods during which the phase-lock is lost. For high values of *G*, the nodes are synchronized, leading to a stable relation between their phases.

**Fig 7:**
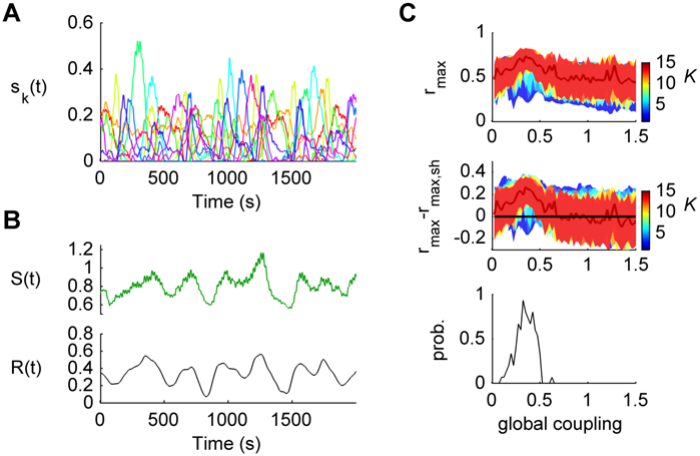
Emergence of transient synchronization patterns. **a)** Temporal evolution of activation strengths of the communities of the anatomically connected heterogeneous Kuramoto model (*G* = 0.2 and *K* = 10). **b)** Temporal evolution of the total community activation strength *S* (*top*) and the order parameter *R* (*bottom*) of the model anatomically connected heterogeneous Kuramoto model (*G* = 0.2 and *K* = 10). The correlation coefficient between *S*(*t*) and *R*(*t*) is 0.82 (p<10^–3^). **c)** *Top*: The largest correlation coefficient *r*_*max*_ (and 95% confidence interval) between the model communities and the empirical communities was calculated for various number of components (*K* = 2, …, 16) as a function of the global coupling. *Middle*: *r*_*max*_ was compared to the expected upper 95% confidence bound of the largest correlation coefficient (*r*_*max*, *perm*_) between the model communities and 10^3^ random permutations of the *n* elements of each empirical communities, for each *K* and each *G*. *Bottom*: Probability that *r*_*max*_> *r*_*max*, *perm*_.

In the following, we tested whether the model can generate the observed transient synchronized clusters, as a result of transitions among the metastable states. This was done by constructing the interaction tensor of the model and applying the NNTF (Fig. 7). We found that fluctuations in the global synchronization result from intermittent activation of synchronized communities (Fig. 7a-b). Moreover, the correlation maxima between the model’s communities and the empirical communities indicate a significant similarity between the model and the empirical community structures, for *G* ranging from 0.15 to 0.5 (Fig. 7c).

In conclusion, the simple model used here emulates several temporal and spatial synchronization characteristics of the human resting-state BOLD activity.

## Discussion

Our results demonstrate that the brain’s spontaneous activity can be decomposed into synchronization networks, or communities, that transiently emerge and dissolve, giving rise to a global synchronization that fluctuates on a characteristic ultra-slow time scale (<0.01Hz). Consistent with our findings, resting-state functional connectivity fluctuations between 0.005 and 0.015 Hz have been reported using sliding window analysis of the Pearson correlation matrix of fMRI signals [19]. We used a combination of phase synchrony measuring and NNTF that allowed us to track the synchronization networks with enhanced temporal resolution and without the need of using sliding windows. We showed that such synchronized communities reoccur in time and across scanning sessions and that they relate to previously defined functional networks or combinations of those networks, including the default mode network and other sensory, motor, and cognitive networks [32].

Here, we demonstrated that the slow variation of synchronization is an emergent collective behavior in an anatomically-constrained network of oscillators. Notably, the model consistently *i*) generates the observed fluctuations of the global synchrony at an order of magnitude lower time scale than the frequencies of the oscillators, *ii*) generates the intermittent appearance of transient synchronized networks, and *iii*) approximates multiple empirical phase statistics within the same parameter range. In particular the model efficiently approximates the mean level of global synchrony, the phase differences distribution, and the distribution of synchronized nodes, for the same parameter range. Nevertheless, the model’s prediction of the pairwise phase relations, given by the PLV matrix, is moderate, albeit highly significant within the parameter range for which the aforementioned statistics are approximated. This imperfect fitting is expected principally because the diffusion imaging tractography we used poorly captures the inter-hemispheric connections and does not provide information about the directionality of the connections [33].

Previous studies have used a version of the Kuramoto model to describe the entrainment between the spontaneous oscillations of distant brain areas [27, 34]. In these studies, it was assumed that neural populations oscillate in the gamma range (>30Hz), since this frequency has been associated to the information processing at the local circuit scale [35]. Opposite to this, here we used a network of slow oscillators, with intrinsic frequencies distributed in the frequency range between 0.04–0.07 Hz—but note that the model robustly approximates the empirical statistics in other low frequency narrow bands within 0.01 and 0.13 Hz. The choice of low frequency oscillators is consistent with recent studies showing that the slow electrical activity and the spontaneous BOLD signal are closely linked and produce similar spatial correlation patterns [36, 37], suggesting a common neural mechanism for both signals. Moreover, it has been shown that the slow components of cortical potentials play an important role in the coordination of large-scale networks [36–38]. Note that the slow oscillators used in the present study, allow us to neglect the effect of conduction delays between the different brain regions, which are orders of magnitude faster—tens of milliseconds [39]—than the periods of the model oscillators. The model presented herein describes the interaction between the mesoscopic dynamics among the different brain regions. Whilst the Kuramoto model is a canonical model that captures the collective synchronization phenomena, it should not be taken as a detailed biophysical model. Nevertheless, the Kuramoto model has been shown to approximate any network of sustained oscillators (limit cycles), as soon as the oscillators interact with a sufficiently weak coupling in order to not destroy the limit cycles, in which case only the phases are perturbed [40]. Thus, it is possible to envision a more biophysically detailed model that, after analysing how phases are perturbed (phase response function, PRF), can be reduced to its phase dynamics—provided that the PRF retains at least the first two Fourier components of the original neural interaction [41]. How the mesoscopic oscillations relate to the microscopic dynamics and to the local connectivity in networks of spiking neurons will be the subject of future investigation. In particular, a more detailed model that produces self-sustained oscillations would be very useful for investigating how oscillation amplitudes affect the phase dynamics [42], a question that is not addressed in the present work.

The findings presented here show a direct link between time-varying functional resting-state connectivity and nonlinear dynamics embedded in the complex large-scale brain network. Time-varying network interactions have been studied in the context of adaptive networks, where the structure and the dynamics can co-evolve through, for example, synaptic plasticity [43]. In contrast, here we showed that temporal synchronization networks emerge from the interplay between nonlinear dynamics and the static network structure. Here, transient synchronization is a result of metastability and it does not require any type of short term plasticity, or other time dependence of the network connections, or even transmission delays. However, temporal variations of couplings that are mediated by synaptic plasticity should not be excluded at faster time scales, which are not accessible with fMRI—in particular, synaptic plasticity might play an important active role in orientating the switching between metastable states. Our results are consistent with a recent study [44] showing that fluctuations of the global level of synchrony in complex modular networks, such as the metabolic and the metropolitan transport networks, result from network topology. Indeed, the brain’s anatomical connectivity has small-world and modular attributes [33] that are thus responsible for the temporal variability of the global synchronization. This topological organization has been related to function, since it might play a key role in the integration of information across functionally segregated brain regions [33]. Along with network topology, heterogeneity is necessary in the generation of multiple synchronized clusters. Heterogeneity in frequency content of fMRI time-series in different brain regions might be related to functional segregation since frequency power is significantly different across functional brain networks [45]. Hence, our study suggests that network topology and heterogeneity are two functionally relevant features, implicated in integration and segregation, which are responsible for the observed time-varying functional connectivity at rest.

Previous work argued that integration and segregation are reconciled in the case of metastability [46]. Consistent with this view, the simple model used here indicates that metastability may underlie the observed transient synchronized clusters for which sets of brain regions engage and disengage in time. This phenomenon does not require noise to produce the transient wandering between synchronization clusters, but it is the result of heterogeneous natural frequencies and nonlinear interactions. Metastability is a functionally relevant scenario since it facilitates the exploration of a larger dynamical repertoire of the brain and allows for the all-around visitation of functional states and dynamic responses to the external world. Indeed, there is evidence that the brain tends to enlarge its repertoire of potential states, as the variance/entropy of BOLD signals positively correlates with chronological age [47]. Furthermore, this repertoire can be flexibly modified to adapt to the cognitive demands during tasks [48, 49]. Synaptic plasticity, neuro-modulation, and input gating may provide a mechanism for the brain system to adjust the exploration of potential states in a context-dependent manner. How these mechanisms interact with large-scale dynamics and metastability is an open question that requires further development of more biophysically realistic models.

## Methods

### Ethics statement

This research was conducted in agreement with the Code of Ethics of the World Medical Association (Declaration of Helsinki) and informed consent was obtained from all subjects before performing the study, in accordance with institutional guidelines. The study design was approved by the local Ethics Committee of Chieti University and the local Ethics Committee of Lausanne.

### Acquisition and pre-processing of fMRI time series

The resting BOLD activity was measured in 24 right-handed healthy young volunteers (15 females, age range 20–31 years). Subjects were informed about the experimental procedures and provided written informed consent. The study design was approved by the local Ethics Committee of Chieti University. Subjects lay in a supine position and viewed a black screen with a centered red fixation point of 0.3 visual degrees, through a mirror tilted by 45 degrees. Each volunteer underwent two scanning runs of 10 minutes in a resting-state condition. Specifically, they were instructed to relax, but to maintain fixation during scanning. The eye position was monitored at 120 Hz during scanning using an ISCAN eye tracker system. Scanning was performed with a 3T MR scanner (Achieva; Philips Medical Systems) at the Institute for Advanced Biomedical Technologies in Chieti, Italy. Functional images were obtained using T2-weighted echo-planar imaging (EPI) with blood oxygenation level-dependent (BOLD) contrast using SENSE imaging. EPIs (TR/TE = 2000/35 ms) comprised 32 axial slices acquired continuously in ascending order covering the entire brain (voxel size = 3×3×3.5 mm^3^). For each scanning run, initial 5 dummy volumes allowing the MRI signal to reach steady state were discarded. The next 300 functional volumes were used for the analysis. A three-dimensional high-resolution T1-weighted image (TR/TE = 9.6/4.6 ms, voxel size = 0.98×0.98×1.2 mm^3^) covering the entire brain was acquired at the end of the scanning session and used for anatomical reference. Initial data pre-processing was performed using the SPM5 software package (Wellcome Department of Cognitive Neurology, London, UK) running under MATLAB (The Mathworks, Natick, MA). The pre-processing steps involved the following: (1) correction for slice-timing differences (2) correction of head-motion across functional images, (3) co-registration of the anatomical image and the mean functional image, and (4) spatial normalization of all images to a standard stereotaxic space (Montreal Neurological Institute, MNI) with a voxel size of 3×3×3 mm^3^. Furthermore, the BOLD time series in MNI space were subjected to spatial Independent Component Analysis (ICA) for the identification and removal of artifacts related to blood pulsation, head movement and instrumental spikes [7]. The BOLD artifact removal procedure was performed using the GIFT toolbox (http://mialab.mrn.org/software/gift/index.html). No global signal regression was performed. Finally, for each recording session (subject and run), we extracted the mean BOLD time series from the 66 brain regions of the brain atlas [33].

### Structural connectivity matrix

The anatomical connectivity matrix was estimated using Diffusion Spectrum Imaging (DSI) data and tractography from five healthy right-handed male human subjects [33]. The experimental design was approved by the local ethics committee at University of Lausanne. The grey matter was subdivided in 33 cortical regions per hemisphere (*n* = 66 areas in total) according to anatomical landmarks (same as for the BOLD signals). This was followed by a further parcellation into 998 regions of interest (ROIs). White matter tractography was used to estimate the fiber tract density connecting each pair of ROIs, averaged across subjects. Anatomical connectivity among the 66 cortical regions was calculated by summing all incoming finat strengths to the target region, and dividing it by its region-dependent number of ROIs, resulting in a non-symmetric connectivity matrix. The normalization by the number of ROIs—which have approximately the same surface on the cortex, i.e. the same number of neurons—is required because neuronal activity is sensitive to the number of incoming fiber per neuron in the target region. The connection of a region to itself was set to 0 in the connectivity matrix for the simulations.

### Phase synchronization

We extracted the phases of the fMRI time series of each of the *n* brain regions (*n* = 66). Following [26], the BOLD signals were first band-pass filtered within the narrowband 0.04–0.07 Hz. This frequency band has been mapped to the gray matter and it has been shown to be more reliable and functionally relevant than other frequency bands [1, 26, 50]. The Hilbert transform (HT) was applied to the filtered BOLD signals to obtain the associated analytical signals. The analytic signal represents a narrowband signal, *a*(*t*), in the time domain as a rotating vector with an instantaneous phase, *φ*(*t*), and an instantaneous amplitude, *A*(*t*), i.e., *a*(*t*) = *A*(*t*)cos(*φ*(*t*)). The phase and the amplitude are given by the argument and the modulus, respectively, of the complex signal *z*(*t*), given by *z*(*t*) = *a*(*t*)+*j*.HT[*a*(*t*)], where *j* is the imaginary unit. Note that narrowband filtering is a requirement for obtaining meaningful phases and envelopes through the HT [26].

The global level of phase synchrony was quantified by the order parameter, *R*(*t*), given by:

$$R(t)=\left|\frac{1}{n}\sum_{i=1}^{n}e^{j\varphi_{i}(t)}\right|$$ (1)

Thus, *R* is the average phase of the system and takes the values 0 and 1 for the completely asynchroneous and completely synchronized cases, respectively. For a finite number *n* of independent phases, the expected value of *R* is equal to *n* ^-1/2^. To quantify the pairwise phase relation between two given brain regions *k* and *l*, we calculated the phase-locking value (PLV), given as:

$$PLV(k,l)=\left|\frac{1}{T}\sum_{t=1}^{T}e^{j\left[\varphi_{k}(t)-\varphi_{l}(t)\right]}\right|$$ (2)

Thus, the phase locking value ranges from 0 (complete phase independence) to 1 (perfect phase locking: *φ*_*k*_(*t*)–*φ*_*l*_(*t*) = const., for all *t*).

### Phase randomized surrogates

Phase shuffled surrogates were used to assess the significance of synchronization. For this, the Fourier transform (FT) of the original signals was first compute and the phase of the Fourier coefficients was substituted with uniformly distributed random numbers while preserving their modulus. Second, the inverse FT was applied to return to the time domain. This procedure effectively randomizes the phases of the signals while preserving the same power spectra as the original series. Specifically, let *x*_*i*_(*t*) be the original BOLD signal from region *i* (*i* = 1, …, *n* and *t* = 1, …, *T*). The discrete Fourier transform of *x*_*i*_ is given by:

$${\tilde{x}}_{i}(k)=\sum_{t=1}^{T}x_{i}(t)e^{-j\frac{2\pi kt}{T}}$$ (3)

Where *j* is the imaginary unit and *k* = 1, …, *T*. A realization of the phase randomized surrogate is given by:

$$x_{i}^{surr}(t)=\frac{1}{T}\sum_{k=1}^{T}\left|{\tilde{x}}_{i}(k)\right|e^{-j\left(\frac{2\pi kt}{T}+\varphi_{r}\right)}$$ (4)

Where *φ*_*r*_ is a random variable uniformly distributed between—π and π. Finally, the surrogate time series were band-pass filtered within the narrowband 0.04–0.07 Hz and the global phase synchronization (order parameter) was computed.

We used these surrogates *i*) to evaluate the global level of phase synchrony (*R*) on the null hypothesis of null synchronization and *ii*) to correct the bias of the PLV matrix by subtracting to each entry of the matrix PLV(*k*, *l*) the expected value when the phases of the time-series *k* and *l* are phase-randomized (estimated using 10^3^ randomizations for each pair (*k*, *l*)).

### Amplitude and phase correlations

We evaluated the contribution of the amplitudes and the phases to the observed correlations among the BOLD signals. For this, we compared the correlation matrix among the narrowband signals, the correlation matrix among the signal amplitudes and the phase-locking matrix of the signal phases. Amplitudes and phases were extracted from the analytic signals, *a*(*t*) = *A*(*t*)cos(*φ*(*t*)), where *A*(*t*) is the instantaneous amplitude and *φ*(*t*) is the instantaneous phase. Phase-locking values were given by Equation (2). The Pearson correlation coefficients were computed for all pairs of narrowband signals and for all pairs of amplitudes of the analytical signals that were transformed by taking logarithm of the squared amplitude envelopes to render the amplitudes statistics more normal before calculating the correlations [51]:

$$r_{signal}(i,j)=\frac{\sum_{t=1}^{T}\left(x_{i}(t)-\langle x_{i}(t)\rangle \right)\left(x_{j}(t)-\langle x_{j}(t)\rangle \right)}{\sqrt{\sum_{t=1}^{T}{\left(x_{i}(t)-\langle x_{i}(t)\rangle \right)}^{2}{\left(x_{j}(t)-\langle x_{j}(t)\rangle \right)}^{2}}},$$ (5)

$$r_{amp}(i,j)=\frac{\sum_{t=1}^{T}\left({\hat{A}}_{i}(t)-\langle {\hat{A}}_{i}(t)\rangle \right)\left({\hat{A}}_{j}(t)-\langle {\hat{A}}_{j}(t)\rangle \right)}{\sqrt{\sum_{t=1}^{T}{\left({\hat{A}}_{i}(t)-\langle {\hat{A}}_{i}(t)\rangle \right)}^{2}{\left({\hat{A}}_{j}(t)-\langle {\hat{A}}_{j}(t)\rangle \right)}^{2}}}$$ (6)

Where *x*_*i*_(*t*) is the original narrowband signal from region *i* (*i* = 1, …, *n* and *t* = 1, …, *T*), *Â* is the square-logarithmic transform of *A*, and <.> denotes the average over time.

For each pair of brain regions, we calculated the narrowband signals’ correlations (*r*_*signal*_), amplitudes’ correlations (*r*_*amp*_) and phase-locking values (PLV), averaged across scanning sessions, and compared them (S1 Fig.). Note that the relation between both *r*_*signal*_ and *r*_*amp*_ and between *r*_*signal*_ and PLV is not linear (S1 Fig.). To quantify how well the interactions among amplitudes and the interactions among phases represent the interactions among signals, we evaluated the uncertainty reduction (Δ) about the narrowband signals’ correlations given the amplitude correlations or given the phase-locking values. This was done by computing the normalized mutual information *MI* between the different measures, given by:

$$\Delta (y)=\frac{MI(r_{signal},y)}{H(r_{signal})}=\frac{1}{H(r_{signal})}\left[H(r_{signal})+H(y)-H(r_{signal},y)\right]$$ (7)

Where *H* denotes the entropy and *y* represents either *r*_*amp*_ or PLV. We found that the uncertainty reduction is slightly higher for the phase interactions than for the amplitude interactions (Δ(PLV) = 0.37; Δ(*r*_*amp*_) = 0.21). When comparing individual scanning sessions we found that Δ(PLV) is significantly (p<10^–10^, *t*-test) higher than Δ(r_amp_). Thus, phase interactions convey more information about the signals’ correlations than do the amplitudes’ correlations. This difference is mainly due to the fact that only high narrowband signals’ correlations are well reflected in the amplitude correlations, while weak and moderate narrowband signals’ correlations are associated to near zero amplitude correlations. Hence, phase interactions are a good description of the interactions of the narrowband signals and, importantly, allow for a time-resolved analysis of interactions, as shown below.

### Time-varying networks

We detected the community structure of temporal synchronization networks by using a non-negative tensor factorization (NNTF) approach, based on the canonical decomposition procedure, which can be seen as a higher-order analogue of the Principal Component Analysis (PCA) [52]. This approach has successfully been applied to detect the community structure of temporal networks [28]. Unlike PCA that uses a vector-based representation, tensor factorization represents the spatial interaction matrices within the network’s nodes as a 3-dimensional space-time tensor and seeks for the most parsimonious decomposition of the tensor onto sum of *simpler* tensors. Tensor factorization preserves the two-dimensional character of spatial interactions (something that is lost when interaction matrices are vectorized in a PCA) and extracts the temporal coherence of the spatial patterns.

### Tensor representation of synchronization networks

A synchronization matrix **Q** was built at each time step *t* (*t* = 1, .., *T*) by calculating the phase difference of each pair of empirical analytic signals (or phase oscillators in the model) and imposing a synchronization threshold that, unless otherwise specified, was equal to π/6, i.e.: Q_*ij*_(*t*) = 1 if |*φ*_*j*_(*t*)–*φ*_*i*_(*t*)|*<*π/6 and Q_*ij*_(*t*) = 0 otherwise (1 *≤ i*, *j ≤ n*). In this way, we obtained a three-dimensional matrix, or tensor **T**, of size *n*×*n*×*T*, with values equal to 0 or 1, given by **T**(*i*, *j*, *t*) = Q_*ij*_(*t*). To eliminate the possibly accidental phase synchronizations, a link (*i*, *j*) was set to zero, for all time steps, if the phases of signals *i* and *j* was such that |*φ*_*j*_(*t*)–*φ*_*i*_(*t*)|<π/6 for less than 20% of the time steps. The tensor **T** contains the topological and temporal information of phase synchronization.

### Tensor decomposition

The tensor **T** was decomposed into *K* rank-one tensors in the form:

$$Τ=\sum_{k=1}^{K}a_{k}\circ b_{k}\circ c_{k}$$ (8)

Where ***a***_***k***_, ***b***_***k***_, and ***c***_***k***_ are vectors of dimension *n*, *n*, and *T*, respectively, and “о” represents the outer product of vectors, defined as **u**o**v** = **u**.**v** ^*T*^, where the superscript *T* denotes the transpose. The aim of the tensor canonical decomposition is to find the set of *factor matrices*, **A** = [***a***_***1***_ … ***a***_***K***_], **B** = [***b***_***1***_ … ***b***_***K***_], and **C** = [***c***_***1***_ … ***c***_***K***_] that best approximates **T** (Fig. 3a). In particular, here we imposed a non-negative constrain on the factor matrices as this provides an additive representation of the tensor in terms of the factors, thus providing a physically meaningful interpretation of the decomposition [53]. The optimization problem is equivalent to minimize the difference between **T** and the approximation, given the non-negative constrains, i.e.:

$$\min{\left\|Τ-{Τ}_{A,B,C}^{approx}\right\|}^{2},\text{with}\;A,B,C\geq 0$$ (9)

Where ‖.‖ represents the Frobenius norm and ${Τ}_{A,B,C}^{approx}=\sum_{k=1}^{K}a_{k}\circ b_{k}\circ c_{k}$. We used the recently proposed block principal pivoting method (www.cc.gatech.edu/~hpark/nmfsoftware.php) to achieve this optimization [54].

Since at each time step *t*, the matrix **Q** is symmetric, we have **A = B**. The community structure of the network and the temporal activation of the different communities are contained in the matrices **A** and **C**, respectively. The column vectors of **A** represent the *K* communities, with *a*_*k*_(*i*) being the associated participation weight of the node *i* in the community *k*. The column vectors of **C** represent the activity level of each community, i.e., *c*_*k*_(*t*) is the activation level of community *k* at time step *t*. The strength of a community *k*, noted *s*_*k*_(*t*), can be calculated as a combination of the level of activation of this community and its total participation weight [28]:$s_{k}(t)=c_{k}(t)\sum_{i=1}^{n}a_{k}(i)$.

Finally, the number of components was selected using the DIFFIT method [55]. This procedure calculates the goodness-of-fit for each *K*, given by $F(K)=1-\left\|Τ-{Τ}_{A,B,C}^{approx}(K)\right\|/\left\|T\right\|$, and detects the number of components *K* after which the function F enters into a plateau by selecting the value that maximize the function: DIFIT(*K*) = [F(*K*)–F(*K*–1)]/F(*K*+1). The selected number of components range between 6–13 for the individual scanning fMRI sessions (median: 9). For simplicity, we chose the median value (*K* = 9) for all scanning sessions. The selected number of components was equal to 14 for the two tensors constructed by concatenating each half of all scanning fMRI sessions, respectively. Finally, in a second analysis we changed the synchronization threshold from π/6 to π/4 (S2 Fig.); the selected number of components using a threshold equal to π/4 was equal to 15 and 16 for the two tensors constructed by concatenating each half of all scanning fMRI sessions, respectively.

### Comparison with Independent Component Analysis

We compared the synchronization communities obtained using the NNTF with the spatial components obtained using spatial Independent Component Analysis (ICA). Spatial ICA is a standard technique that clusters the data into maximally spatially independent patterns of coherent fMRI activity [2, 32], i.e., voxels belonging to a given ICA pattern have higher temporal correlations among themselves than with voxels belonging to other ICA patterns. Here we used the GIFT toolbox to perform the ICA decomposition for each scanning session. The estimation of the number of independent components (ICs) was performed using the minimum description length criterion [2]. Each IC consisted of a spatial pattern of activity (z-score) and a corresponding time-course of the spatial pattern. Self-organizing group ICA (sogICA) [56] was used to average the ICs extracted from single scanning fMRI datasets. Fourteen ICs were identified, ICs were for the most part consistent, and were present in at least 75% of the subjects [32]. The ICA patterns at the voxel level were down-sampled onto the same parcellation of 66 cortical regions used in the present study by averaging the results among the voxels belonging to each cortical region. The ICA patterns were associated to different functional networks which are often seen in resting-state fMRI activity and that consist of sets of brain regions known to be engaged in sensory-motor or cognitive function, called resting-state networks (RSNs) [32]. The RSNs include the default mode, core, somatomotor, dorsal/ventral attention, vision, auditory, self-referential, language, cognitive control, and working memory networks. For further details about the ICA procedure see [32].

For comparison, the synchronization communities and the ICA-based RSNs were converted to 66-dimensional binary vectors by imposing a threshold, equal to 0.1, above which the elements were set to 1 and, otherwise, they were set to 0. As a measure of similarity between the binarized synchronization communities and the binarized ICA RSNs we used the Jaccard index. The Jaccard index is defined as the proportion of nonzero coordinates that are equal in two given binary vectors.

### Kuramoto model

We considered a simple model of a network of *n* coupled phase oscillators, in which connections are determined by the anatomical connectivity matrix that was estimated using DSI (*n* = 66). The Kuramoto model is considered a canonical model of synchronous oscillations in many systems [57]. This model assumes that the oscillators interact with each other through their phase differences. Let *φ*_*i*_(*t*) be the phase of the *i*-th oscillator (*i* = 1, …, *n*) at time *t*. The time evolution of the phases is governed by the following set of coupled differential equations:

$$\frac{d\varphi_{i}}{dt}=\omega_{i}+G\sum_{j=1}^{n}C_{ij}^{\text{anat}}\sin\left(\varphi_{j}-\varphi_{i}\right)$$ (10)

Where *ω*_*i*_ is the natural frequency of the *i*-th oscillator, **C** ^anat^ is the *n*×*n* anatomical coupling matrix, and the parameter *G* represents the global coupling strength. The interaction between two given oscillators *i* and *j* is modulated by the sinus of the phase difference, sin(*φ*_*j*_—*φ*_*i*_). Such interaction tends to synchronize the oscillators since an oscillator lagging behind another one (*φ*_*j*_—*φ*_*i*_>0) is speed up, whereas an oscillator leading another (*φ*_*j*_—*φ*_*i*_<0) is slow down. The model was numerically integrated using the Euler’s method with a time step equal to 0.01, equivalent to 10ms. The total number of simulation steps was 12.10^5^ and the first 5.10^5^ steps were discarded to remove the transient period.

In addition, we considered the effect of adding noise to the anatomically-connected homogeneous Kuramoto model. This model (called model 4 in the following) assumes that all the oscillators have the same intrinsic frequency *ω*_0_ = 0.05 Hz and receive independent uncorrelated white noise. The time evolution of the phases is governed by the following set of coupled stochastic differential equations:

$$\frac{d\varphi_{i}(t)}{dt}=\omega_{0}+\xi_{i}(t)+G\sum_{j=1}^{n}C_{ij}^{\text{anat}}\sin\left(\varphi_{j}(t)-\varphi_{i}(t)\right)$$ (11)

Where *i* = 1, …, *n*, **C** ^anat^ is the *n*×*n* anatomical coupling matrix, scaled by the global coupling strength *G*, and *ξ*_*i*_ is uncorrelated white noise, i.e., <*ξ*_*i*_(*t*)> = 0 and <ξ_*i*_(*t*)*ξ*_*j*_(*t’*)> = σ^2^ *δ*(*t’–t*)*δ*_*ij*_, where σ is the noise amplitude. Noisy fluctuations can be interpreted as the modification of the phase of each oscillator due to the underlying neural noise, which is a consequence of random fluctuations of the background activity and inherent to neural networks of finite size [58].

### Distribution fitting

We use the Kullback-Liebler divergence, *D*_*KL*_, to evaluate how well the Kuramoto model describes the empirical probability distribution of phase difference, Pr(Δφ), and the empirical probability distribution of the number of synchronized pairs, Pr(*N*). Given an observed (empirical) probability distribution *g* and a model probability distribution *f*, *D*_*KL*_ measures the dissimilarity between the two distributions. It is defined to be:

$$D_{KL}(g,f)=\int g(x)\log\left(\frac{g(x)}{f(x)}\right)\;dx$$ (12)

The smaller *D*_*KL*_ means that the model distribution *f* is closer to the empirical distribution *g*. We used the inverse of *D*_*KL*_ as the goodness-of-fit of the model.

## Supporting Information

S1 Fig Amplitude interactions vs. phase interactions. Comparison between all pairwise correlations among the narrowband signals (*r*_*signal*_) and the corresponding correlations among the signal amplitudes (*r*_*amp*_) and phase-locking values among phases (PLV). The top panel shows the distribution of *r*_*signal*_ and the right panel shows the distributions of *r*_*amp*_ and PLV, respectively. The dots represents all possible *n*(*n*-1)/2 pairwise interactions for the corresponding measures.
(TIF)

S2 Fig Effect of the synchronization threshold. Two synchronization tensors were constructed by concatenating each half of all scanning sessions and by applying a synchronization threshold. For a synchronization threshold equal to π/4, the selected number of components was equal to 15 and 16 for the first and second half-dataset tensors, respectively. The selected number of components was equal to 14 for the two tensors constructed by applying a synchronization threshold equal to π/6, for both half-datasets, as previous. The synchronization communities obtained using different synchronization thresholds (equal to π/6 or π/4) were compared by computing the correlation coefficient between the respective vectors ***a***_***k***_. The correlation similarity matrix between synchronization communities is shown for the first half of the data (*left*) and the second half of the data (*right*). Similar community patterns are consistently found for different synchronization thresholds.
(TIF)

S3 Fig Deterministic vs. stochastic dynamics. We compared the similarity between the empirical statistics and the statistics generated by the stochastic anatomically-connected homogeneous Kuramoto model (model 4). For each pair of parameters {*G*, σ}, 50 stochastic realizations of model 4 were simulated and the averaged (and 95% confidence intervals) similarity value was stored for each statistic. **a)** Averaged value of the order parameter, <*R*>. The noise prevents the system from reaching synchronization if the noise amplitude σ is sufficiently large compared to *G*. **b)** Similarity (1/ *D*_*KL*_) between the phase differences distribution, Pr(Δφ), of the empirical data and model 4. **c)** Similarity (1/ *D*_*KL*_) between the distribution of the number *N* of synchronized pairs of the empirical data and model 4. **d)** Agreement (correlation) between the empirical PLV matrix and the models’ PLV matrices. The red area indicates the 95% confidence interval of the sample Pearson correlation coefficient. All similarity and agreement values were normalized to the maximum similarity/agreement obtained with model 1. **e)** Peak of the power spectrum of the order parameter *R*. The temporal variations of the order parameter are much slower than in model 1.
(TIF)

S4 Fig Model prediction of phase statistics for different narrow frequency bands. The empirical statistics were calculated for four different frequency bands (0.01–0.04 Hz; 0.04–0.07 Hz; 0.07–0.10 Hz; 0.10–0.13 Hz) and were compared to the statistics generated by the anatomically connected Kuramoto model with intrinsic frequencies estimated from the corresponding band-filtered fMRI data. **a)** Averaged value of the order parameter, <*R*>. **b)** Agreement (correlation) between the empirical PLV matrix and the model’s PLV matrix. **c)** Similarity (1/*D*_*KL*_) between the phase differences distribution, Pr(Δφ), of the empirical data and the model. **d)** Similarity (1/*D*_*KL*_) between the distribution of the number *N* of synchronized pairs, Pr(*N*), of the empirical data and the model.
(TIF)

S1 Table Names and abbreviations of the brain regions considered in the human connectome from Hagmann et al. (2008) [33] (in alphabetical order). (DOC)

S1 Dataset BOLD fMRI time-series from a total of 48 scanning sessions, from 24 healthy human subjects, of 600s (sampled in *T* = 300 frames) during resting-state condition. Each volunteer underwent two scanning runs (blocks). For each recording session (subject and run), BOLD time series were averaged over the corresponding 66 brain regions of the brain atlas. Each file *subXblockY*.*txt* contains the time series of all 66 brain regions from subject *X* and run *Y*. The labels of the brain regions are contained in file *ROIs_Labels*.*txt*.
(ZIP)
