# Supplementary figures and images for: Resting-State Temporal Synchronization Networks Emerge from Connectivity Topology and Heterogeneity
Source: PLoS Comput Biol. 2015 Feb 18;11(2):e1004100. doi: 10.1371/journal.pcbi.1004100 (PMC4333573; doi:10.1371/journal.pcbi.1004100)

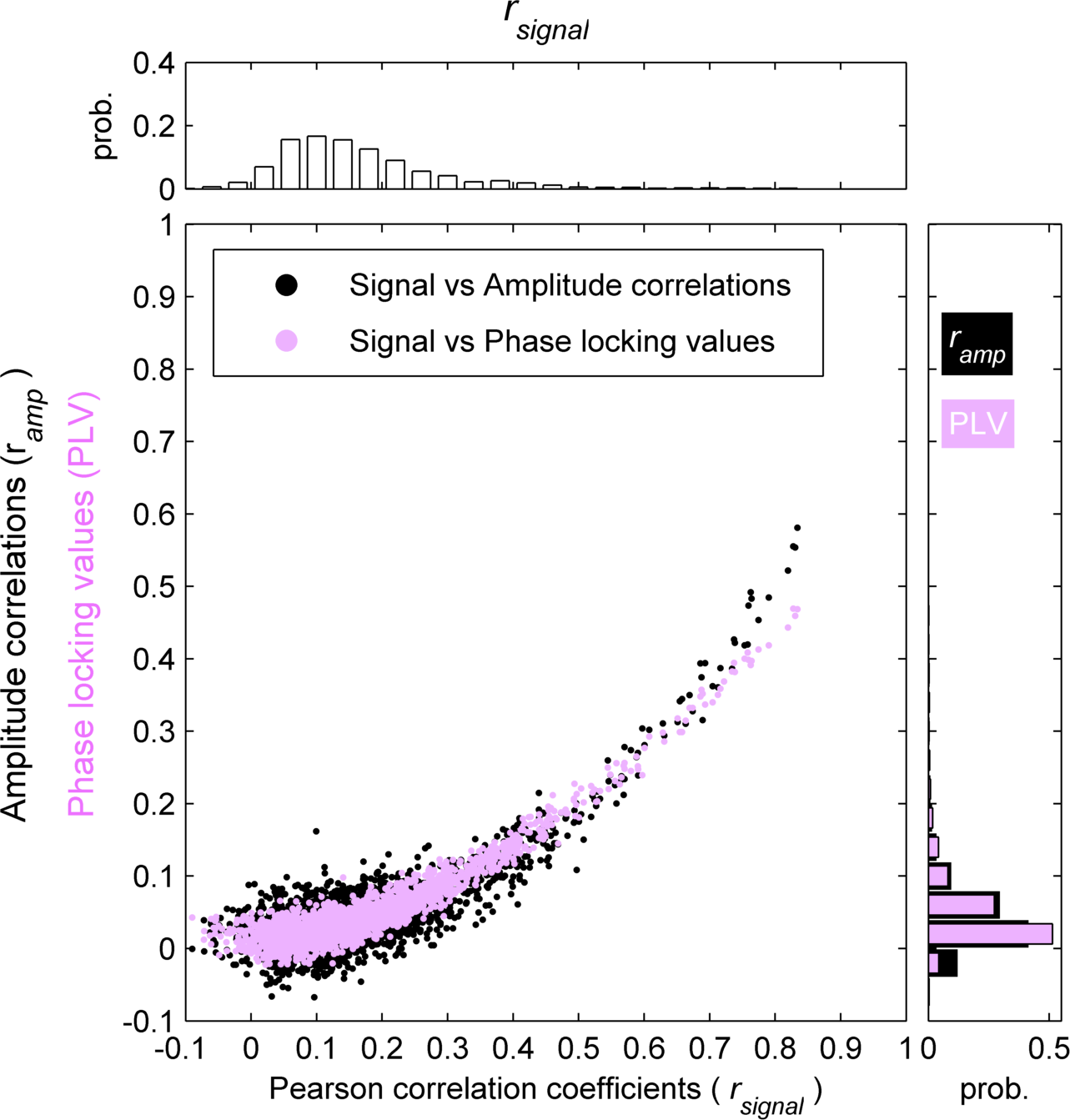

Supplement: S1 Fig — Comparison between all pairwise correlations among the narrowband signals (r signal) and the corresponding correlations among the signal amplitudes (r amp) and phase-locking values among phases (PLV). The top panel shows the distribution of r signal and the right panel shows the distributions of r amp and PLV, respectively. The dots represents all possible n(n-1)/2 pairwise interactions for the corresponding measures. (TIF) [file pcbi.1004100.s001.tif]

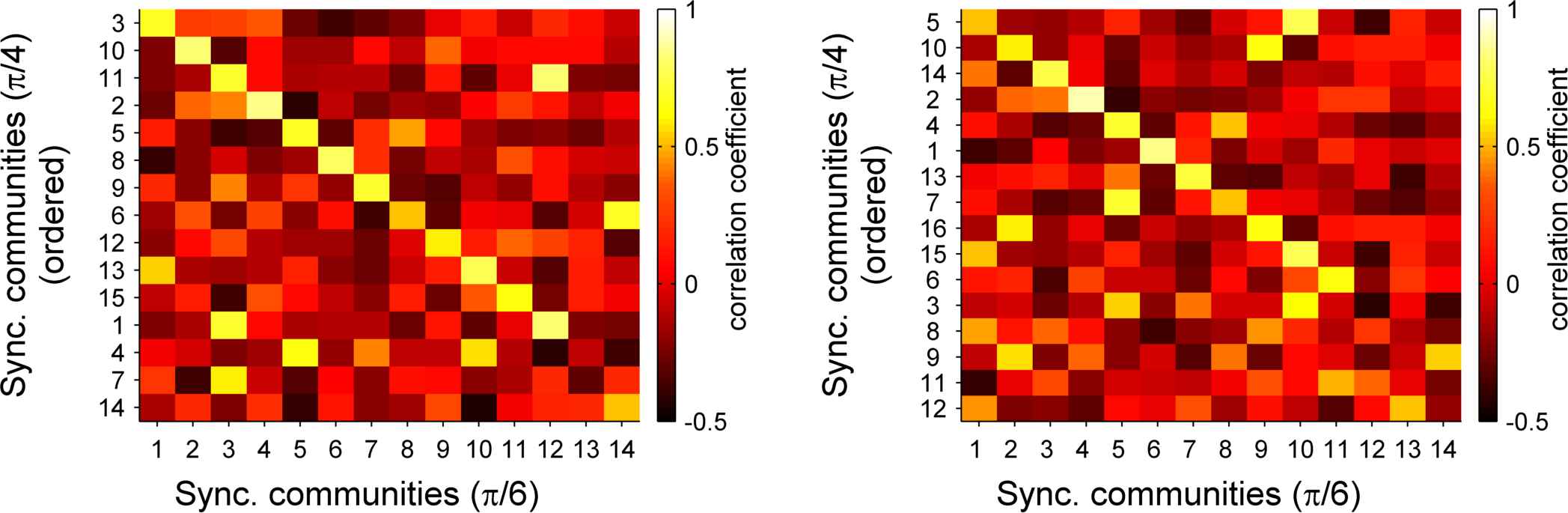

Supplement: S2 Fig — Two synchronization tensors were constructed by concatenating each half of all scanning sessions and by applying a synchronization threshold. For a synchronization threshold equal to π/4, the selected number of components was equal to 15 and 16 for the first and second half-dataset tensors, respectively. The selected number of components was equal to 14 for the two tensors constructed by applying a synchronization threshold equal to π/6, for both half-datasets, as previous. The synchronization communities obtained using different synchronization thresholds (equal to π/6 or π/4) were compared by computing the correlation coefficient between the respective vectors a k. The correlation similarity matrix between synchronization communities is shown for the first half of the data (left) and the second half of the data (right). Similar community patterns are consistently found for different synchronization thresholds. (TIF) [file pcbi.1004100.s002.tif]

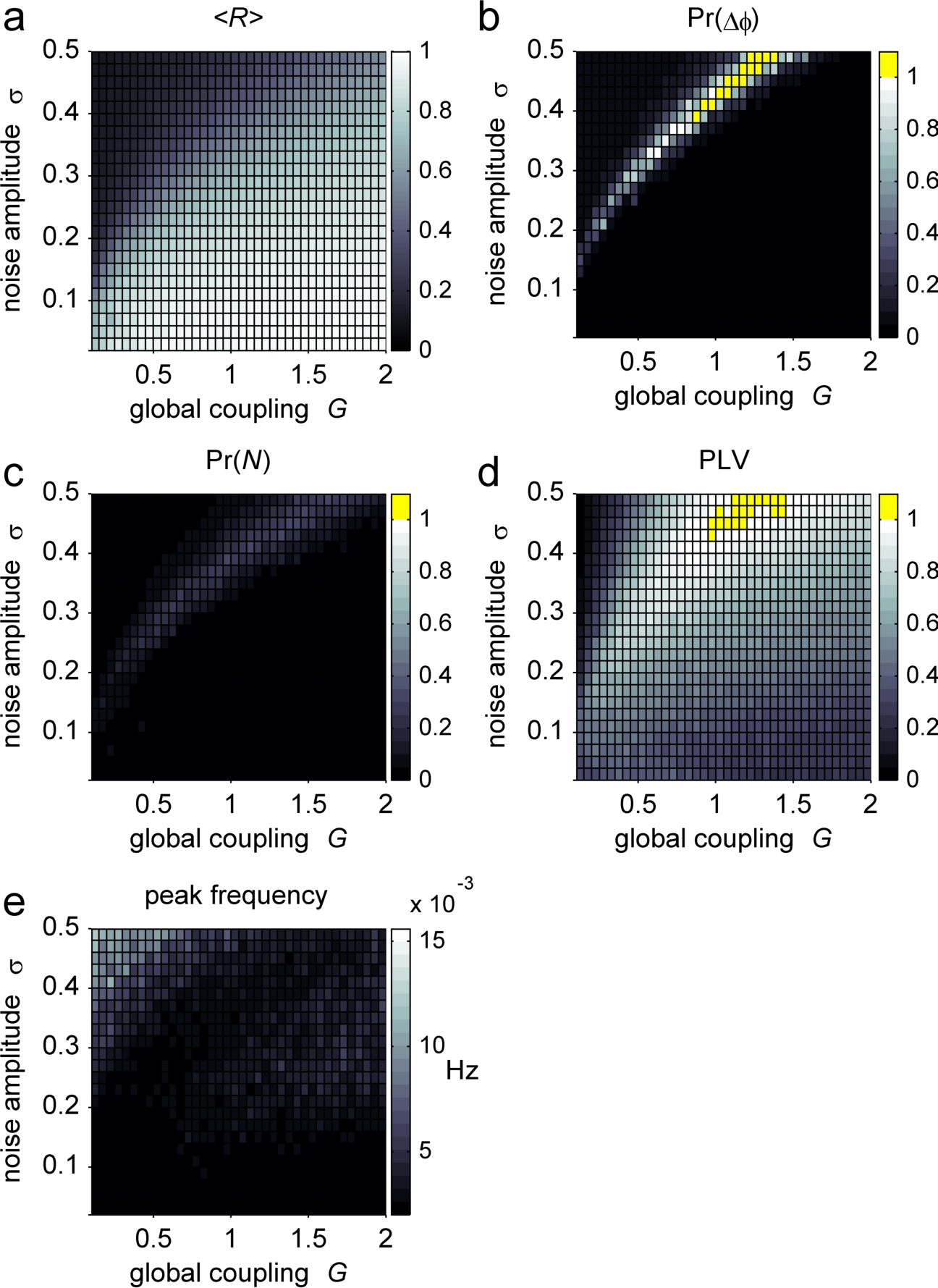

Supplement: S3 Fig — We compared the similarity between the empirical statistics and the statistics generated by the stochastic anatomically-connected homogeneous Kuramoto model (model 4). For each pair of parameters {G, σ}, 50 stochastic realizations of model 4 were simulated and the averaged (and 95% confidence intervals) similarity value was stored for each statistic. a) Averaged value of the order parameter, . The noise prevents the system from reaching synchronization if the noise amplitude σ is sufficiently large compared to G. b) Similarity (1/ D KL) between the phase differences distribution, Pr(Δφ), of the empirical data and model 4. c) Similarity (1/ D KL) between the distribution of the number N of synchronized pairs of the empirical data and model 4. d) Agreement (correlation) between the empirical PLV matrix and the models’ PLV matrices. The red area indicates the 95% confidence interval of the sample Pearson correlation coefficient. All similarity and agreement values were normalized to the maximum similarity/agreement obtained with model 1. e) Peak of the power spectrum of the order parameter R. The temporal variations of the order parameter are much slower than in model 1. (TIF) [file pcbi.1004100.s003.tif]

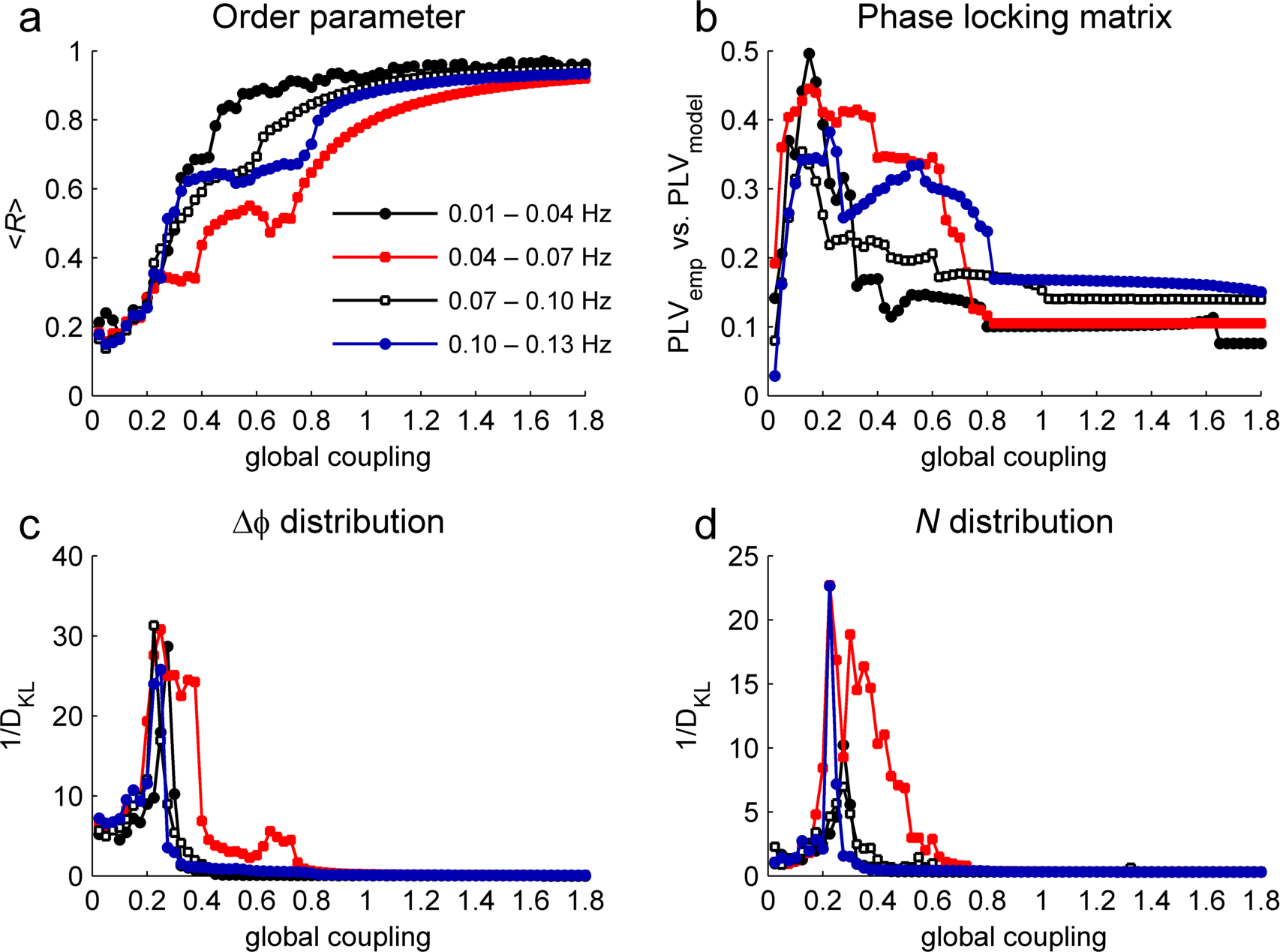

Supplement: S4 Fig — The empirical statistics were calculated for four different frequency bands (0.01–0.04 Hz; 0.04–0.07 Hz; 0.07–0.10 Hz; 0.10–0.13 Hz) and were compared to the statistics generated by the anatomically connected Kuramoto model with intrinsic frequencies estimated from the corresponding band-filtered fMRI data. a) Averaged value of the order parameter, . b) Agreement (correlation) between the empirical PLV matrix and the model’s PLV matrix. c) Similarity (1/D KL) between the phase differences distribution, Pr(Δφ), of the empirical data and the model. d) Similarity (1/D KL) between the distribution of the number N of synchronized pairs, Pr(N), of the empirical data and the model. (TIF) [file pcbi.1004100.s004.tif]
