# Supplementary material for: Resting-State Temporal Synchronization Networks Emerge from Connectivity Topology and Heterogeneity
Source: PLoS Comput Biol. 2015 Feb 18;11(2):e1004100. doi: 10.1371/journal.pcbi.1004100 (PMC4333573; doi:10.1371/journal.pcbi.1004100)
Supplement: S1 Table — (DOC) [file pcbi.1004100.s005.doc]

| Abbreviation | Brain region |
| --- | --- |
| **BSTS** | **Bank of the superior temporal sulcus** |
| **CAC** | **Caudal anterior cingulate cortex** |
| **CMF** | **Caudal middle frontal cortex** |
| **CUN** | **Cuneus** |
| **ENT** | **Entorhinal cortex** |
| **FP** | **Frontal pole** |
| **FUS** | **Fusiform gyrus** |
| **IP** | **Inferior parietal cortex** |
| **ISTC** | **Isthmus of the cingulate cortex** |
| **IT** | **Inferior temporal cortex** |
| **LING** | **Lingual gyrus** |
| **LOCC** | **Lateral occipital cortex** |
| **LOF** | **Lateral orbitofrontal cortex** |
| **MOF** | **Medial orbitofrontal cortex** |
| **MT** | **Middle temporal cortex** |
| **PARC** | **Paracentral lobule** |
| **PARH** | **Parahippocampal cortex** |
| **PC** | **Posterior cingulate cortex** |
| **PCAL** | **Pericalcarine cortex** |
| **PCUN** | **Precuneus** |
| **POPE** | **Pars opercularis** |
| **PORB** | **Pars orbitalis** |
| **PREC** | **Precentralgyrus** |
| **PSTC** | **Postcentralgyrus** |
| **PTRI** | **Pars triangularis** |
| **RAC** | **Rostral anterior cingulate cortex** |
| **RMF** | **Rostral middle frontal cortex** |
| **SF** | **Superior frontal cortex** |
| **SMAR** | **Supramarginalgyrus** |
| **SP** | **Superior parietal cortex** |
| **ST** | **Superior temporal cortex** |
| **TP** | **Temporal pole** |
| **TT** | **Transverse temporal cortex.** |

**Table 1.** Names and abbreviations of the brain regions considered in the human connectome from Hagmann et al. (2008) (in alphabetical order).
